# Supplementary material for: Sedimentary and reservoir characterization of wandering braided river: a case of the Xiashihezi Formation 1 member in Shilijiakhan west zone, Hangjinqi, Northern Ordos Basin
Source: PLoS One. 2025 Sep 8;20(9):e0329942. doi: 10.1371/journal.pone.0329942 (PMC12416704; doi:10.1371/journal.pone.0329942)
Supplement: Attachment — Submitted filename: comments.docx [file pone.0329942.s005.docx]

This paper presents a study of the sedimentary microfacies of the Xiashihezi Formation 1 Member in the Shiligahan area, situated in the northern Ordos Basin. By using core, logging, and 3D seismic data, the sedimentary environment and sedimentary microfacies were successfully identified. The study area was thus determined to be a wandering, shallow braided river sedimentary system. Through calibration and verification among lithofacies, logging facies, and seismic facies, a comprehensive characterization of the plane distribution of braided channels and their internal microfacies in the Xiashihezi Formation 1 Member was achieved. Further analysis of core observation, thin sections, physical properties, and mercury injection curves, particularly through the study and evaluation of pore structure, identified the microscopic characteristics of the reservoir. By integrating macro- and micro-level perspectives, this study elucidates the influence of sedimentation on reservoir development. The article's theme is evident, its structure is logical, and its findings are both novel and practically applicable. Accordingly, I propose that it could be slightly revised. Further detailed comments are provided below:

1. The grammar/English writing of the manuscript can be further improved.

2. The geographic location and other information description of the area should be added in the "geological background" section.

3. In Section 2.2, "The plane distribution of sedimentary microfacies in the Xiashihezi Formation 1 Member was finally obtained by mutual calibration and constraint between the attribute of maximum trough amplitude and the single well sedimentary microfacies, the connected well sedimentary microfacies [21]." And " These conditions were also conducive to the occurrence of dissolution during the diagenetic stage, ultimately forming high-quality reservoirs with favourable physical properties”, Additional basis is needed, such as more references.

4. The legend text in Figure 1 of the paper needs to be adjusted, some fonts need to be enlarged, and some layouts are not standardized enough.

5.The amplitude attribute in figure 6.a of the paper lacks color legend.

6. Only layer and sublayer information appears in Figure 3. It is recommended to complete the complete System, Formation and other information.

7. The corresponding well number can be marked in the sedimentary microfacies type, logging mode and seismic response characteristics in Figure 4.

8.The reference format is not standardized and consistent. Please revise carefully according to the requirements of the journal.
