# Supplementary material for: Sedimentary and reservoir characterization of wandering braided river: a case of the Xiashihezi Formation 1 member in Shilijiakhan west zone, Hangjinqi, Northern Ordos Basin
Source: PLoS One. 2025 Sep 8;20(9):e0329942. doi: 10.1371/journal.pone.0329942 (PMC12416704; doi:10.1371/journal.pone.0329942)
Supplement: Attachment — Submitted filename: reply to plos one.docx [file pone.0329942.s007.docx]

PONE-D-24-37259R1

Sedimentary and reservoir characterization of wandering braided river: a case of the Xiashihezi Formation 1 Member in Shilijiakhan west zone, Hangjinqi, Northern Ordos Basin

Doctor Hongtao Li

Dear Dr. Li,

We've checked your submission and before we can proceed, we need you to address the following issues:

1. Can you please upload. This will be used in the production process if your manuscript is accepted. Please amend the file type for the file showing your changes to Revised Manuscript w/tracked changes. Please follow this link for more information: <http://blogs.PLOS.org/everyone/2011/05/10/how-to-submit-your-revised-manuscript/>

Reply：an additional copy of your revised manuscript that does not contain any tracked changes or highlighting as your main article file already was upload, whose word name is “Shilijiahan reservoir_gai1_revision1_accept all revision.docx”. you can see it in attach files 3.

2. Thank you for updating your data availability statement. You note that your data are available within the Supporting Information files, but no such files have been included with your submission. At this time we ask that you please upload your minimal data set as a Supporting Information file, or to a public repository such as Figshare or Dryad.

Please also ensure that when you upload your file you include separate captions for your supplementary files at the end of your manuscript.

As soon as you confirm the location of the data underlying your findings, we will be able to proceed with the review of your submission.

Reply: I uploaded the original data of the paper in the supporting information, and also uploaded the minimum data set as a Supporting Information file in Zenodo. website is <https://doi.org/10.5281/zenodo.14684491>.

The uploaded information is shown in the following two figures
