# Supplementary material for: Sedimentary and reservoir characterization of wandering braided river: a case of the Xiashihezi Formation 1 member in Shilijiakhan west zone, Hangjinqi, Northern Ordos Basin
Source: PLoS One. 2025 Sep 8;20(9):e0329942. doi: 10.1371/journal.pone.0329942 (PMC12416704; doi:10.1371/journal.pone.0329942)
Supplement: Attachment — Submitted filename: reply to Journal Requirements .docx [file pone.0329942.s008.docx]

Journal Requirements:

Please review your reference list to ensure that it is complete and correct. If you have cited papers that have been retracted, please include the rationale for doing so in the manuscript text, or remove these references and replace them with relevant current references. Any changes to the reference list should be mentioned in the rebuttal letter that accompanies your revised manuscript. If you need to cite a retracted article, indicate the article’s retracted status in the References list and also include a citation and full reference for the retraction notice.
Reply: the references are complete and correct, and an additional reference has been added.

Additional Editor Comments:

Editorial comments to improve the quality and presentation:
1. Please incorporate the range of the petrophysical parameters in the abstract.

Reply：According to the requirements, add petrophysical parameters such as reservoir porosity and average permeability in the abstract.
2. The sentences presented in the abstract is too long, break them and make a simple representation.

Reply：Following the suggestion, the long sentence has been revised to be as short as possible.
3. Please add suitable keywords.

Reply：Added 6 keywords.
4. In Fig. 6a please mention the name of the parameter presented with a color.

Reply：In Figure 6.a, the names of geophysical attributes have been added to the color scale.
5. Please check the axis title of Fig. 10a.

Reply：Modified the vertical axis title in Figure 10a
6. If possible, kindly add a dendrogram analysis based on the hierarchical cluster analysis. You may follow the article "Mukherjee, B., Sain, K. Vertical lithological proxy using statistical and artificial intelligence approach: a case study from Krishna-Godavari Basin, offshore India. Mar Geophys Res 42, 3 (2021). https://doi.org/10.1007/s11001-020-09424-8". Otherwise in the discussion section you can add a few lines and make a recommendation for future researchers to consider this analysis.

Reply：In the discussion section, we add a few lines and make a recommendation for future researchers to consider hierarchical clustering analysis (HCA) analysis. an additional reference of Mukherjee has been added.
